# Effects of acupuncture for cancer pain and quality of life – a case series

**DOI:** 10.1186/1749-8546-8-15

**Published:** 2013-07-30

**Authors:** Sivarama Prasad Vinjamury, Ju-Tzu Li, Eric Hsiao, Calen Huang, Cheryl Hawk, Judith Miller, Yuhong Huang

**Affiliations:** 1 Department of Fundamental Principles, Southern California University of Health Sciences, California, USA; 2 School of Medicine, China Medical University, Taichung, Taiwan; 3 Department of Research, Logan College of Chiropractic/University, Chesterfield, MO, USA; 4 United Health Group, Santa Ana, CA, USA

## Abstract

**Background:**

Many cancer patients seek complementary and alternative medicine (CAM) including acupuncture to manage their cancer-related symptoms or side effects of treatments. Acupuncture is used to manage cancer pain and improve quality of life (QoL). This study aimed to conduct a preliminary study on a case series to evaluate the feasibility of acupuncture for treating cancer pain and to collect preliminary data on the effectiveness of acupuncture in treating cancer pain and improving QoL.

**Methods:**

A semi-standardized acupuncture treatment comprising one to three treatment sessions (20–30 minutes per session) per week for 8 weeks was provided by four licensed acupuncturists, who had more than 5 years of clinical experience, at the University Health Center. The European Organization for Research and Treatment of Cancer Quality of Life Questionnaire (EORTC QLQ-C3) and a visual analogue scale (VAS) for pain rating were used as the outcome measures to assess pain and QoL. Data were collected at baseline, immediately after 2, 4, 6, and 8 weeks of treatment and at 4 weeks after treatment completion (week 12).

**Results:**

Two males and five females with a median age of 66 years (range: 44–71 years) completed the study. For the VAS, the percentage of improvement ranged between 18% and 95%. The baseline mean raw score was reduced from 51 mm to 36 mm at the end of week 8 and to 23 mm at the end of week 12. The percentage of overall QoL improvement ranged between 20% and 100%. The mean raw score for QoL improved with time. The baseline score was increased from 55 to 69 at the end of treatment (week 8) and to 73 after the follow-up (week 12).

**Conclusions:**

This pilot study on a case series showed that acupuncture might be beneficial for reducing pain and improving QoL in cancer patients.

## Background

Cancer is the second leading cause of mortality in the United States [1], and 41.24% of men and women born in the United States will be diagnosed with some type of cancer during their lifetime [2]. Although modern therapies for cancer have improved life expectancy, the management of this complex disease [3] and improvement of quality of life (QoL) of patients, especially in managing cancer-related pain, are still limited. Cancer pain is frequently reported among patients, with 53% of patients experiencing pain, and one-third experiencing severe to moderate levels of acute or chronic pain [4]. The occurrence of pain in cancer patients increases the risk for psychological disorders (*e.g.*, anxiety, depression, and suicidal ideation) [5], that distract patients from their daily activities (*e.g.*, ability to concentrate) [6]. In addition, the cause of pain is not limited to the physiological etiology, but also arises through side effects of standard treatment modalities like drugs, surgery, radiotherapy, and chemotherapy [3].

Opioids are currently used in cancer pain management [7]. The World Health Organization has described a three-step analgesic ladder on how to effectively administer opioids for chronic cancer pain, with the main goal being to prohibit pain by giving high doses of the drugs around-the-clock [8,9]. However, up to 20% of patients are resistant to opioids [10], and of those who are not resistant, many choose not to have treatments that involve narcotic substances because of the various side effects. The common short-term side effects are constipation, sedation, sleep disturbances, nausea, and vomiting [11]. Some patients prefer to endure the pain rather than continue to use opioids [12]. For clinicians, the most concerning side effect is respiratory depression, especially in high-risk patients, such as elderly patients, obese patients, patients with a history of sleep apnea, and patients with impaired pulmonary, renal, cardiac, or hepatic function [11]. Long-term administration of opioids can induce tolerance [13], endocrine dysfunction [14], and multisystem adverse effects [15].

Complementary and alternative medicine (CAM) is currently used in conjunction with standard cancer treatments, with 66.5% of cancer survivors reporting use of CAM, 43.3% of those reported used within one year [16]. Regarding the range, 25–84% of cancer patients seek CAM at least once during their treatments to manage symptoms or side effects [17]. Acupuncture studies have shown potential in alleviating pain and functional disorders associated with specific types of cancers [15]. Although acupuncture was found to have analgesic effects, there are few rigorous studies to support its efficacy [18]. Acupuncture can boost immunity by increasing the numbers of leukocytes, B-cells, immunoglobulins, and erythrocytes, as well as the activity of NK cells [19-21]. It can also alleviate the side effects of chemotherapy or radiotherapy [22], and treat depressive symptoms with equivalent or better efficacy than conventional antidepressants, such as amitriptyline, maprotiline, and mianserin [23,24]. Research on cancer pain is seldom addressed in CAM institutions [25], although some case series are available to warrant investigation [26]. This study aimed to conduct a case series study to investigate the effects of acupuncture on cancer patients recruited from a CAM institution.

## Methods

This study was registered on November 15, 2006 at ClinicalTrial.gov with the registration number NCT00401063.

### Participant recruitment

Over a period of 12 months, flyers were distributed to the community, including 20 local MDs, three oncologists, two cancer support groups, five chiropractors and acupuncturists, 10–15 local businesses, two local libraries, and the local YMCA. In addition, electronic versions of the flyers were periodically distributed to the campus community, comprising approximately 700 people.

This study incorporated specific inclusion and exclusion criteria. The participants were older than 18 years, and of either sex. The participants had to have a confirmed diagnosis of cancer by an oncologist, and a baseline pain score of 3 or more on a 0–10 rating scale. The participants’ pain was evaluated as the results of underlying cancer or cancer treatments. The participants were ambulatory, had platelet counts of 50,000 or greater, and obtained permission from their physicians to participate in the study. Participants were excluded from the study if they had received acupuncture treatment in the past 4 weeks, if they were unable to obtain permission from their treating physicians, if they were unwilling to sign informed consent, or if they were involved in any current litigation. They were also excluded if they were simultaneously infected with HIV/hepatitis B virus, if they had neutropenia defined as an absolute neutrophil count of <1000/mL, or if they had any kind of bleeding disorders where the platelet count was <50,000.

The Southern California University of Health Sciences Institutional Review Board approved the study. It was also registered at the clinicaltrials.gov website. All eligible participants signed an informed consent form prior to clinical screening.

### Study intervention

In this case series study, the acupuncture treatment was semi-standardized for all patients, and based on the type of cancer and the patient’s condition. However, the points *Zusanli* (ST36) and *Sanyinjiao* (SP6) were common to all patients, because *qi* and *yin* will be deficient in all cancer patients, regardless of the type of cancer [24]. The frequency of the treatments varied from one to three treatment sessions per week for 8 weeks. Four licensed acupuncturists, who had more than 5 years of clinical experience, provided the acupuncture treatments. Same clinician treated each participant throughout the study period. Seirin® acupuncture needles of 0.25 mm in diameter and 40 mm in length were used. Each treatment session lasted 20–30 minutes. Electroacupuncture was optional, and was based on the participant’s need as determined by the acupuncturist. All the treatments were provided at the University Health Center at Southern California University of Health Sciences.

### Outcome measures

The European Organization for Research and Treatment of Cancer Quality of Life Questionnaire (EORTC QLQ-C3) and a visual analogue scale (VAS) for pain were used in this study. Both of these measures were shown to be valid and reliable for cancer patients [27-29]. The EORTC QLQ-C3 is a 30-item questionnaire used to assess health-related QoL, which includes five functional scales (physical, role, emotional, cognitive, and social) and eight single-item symptom scales (fatigue, nausea and vomiting, pain, dyspnea, insomnia, appetite loss, constipation, and diarrhea). All functional and symptom scales are 4-point scales (*e.g.*, 1=not at all to 4=very much) with a total range from 0–100. QoL was assessed by a 7-point scale from “very poor” to “excellent”. The descriptions of the scores are as follows: high functional scale score represents a high and healthy level of functioning; high general health status represents a high QoL; high symptom scale score represents a high level of symptomatology. A standard VAS was used to evaluate the participant’s perception of pain. Each participant was provided with a horizontal 100-mm line anchored with the descriptions “no pain” and “worst pain” at each end. The participants marked the line at their current levels of pain, and the evaluator used a ruler to measure the patients’ marks. All participants were asked to complete both outcome measures at the beginning of the study, as well as at the end of weeks 2, 4, 6, and 8. The participants filled in the forms prior to their treatments at each time-point. There was also an on-site follow up evaluation at 4 weeks after the last treatment (end of week 12). No information on participant–provider interactions or patient expectations was collected.

### Data analysis

The demographic characteristics of the patients were described using the mean and standard deviation (SD). The percentages of improvement from baseline in the VAS, and EORTC QLQ and general health status/QoL at the end of week 8 and the end of week 12 (after follow-up) were calculated for individual cases. The percentages were calculated by dividing the difference between the baseline and endpoint scores by the baseline score. The VAS and fatigue and general health status within the EORTC QLQ were presented as the mean ± SD. Values of *P*<0.05 were considered statistical significant. A longitudinal regression analysis (random mixed-effects model) was also performed on an exploratory basis for each outcome measure to describe the overall trend and the rate of change over time (considering variations in the baseline values of all measurements) starting from baseline.

## Results

Twenty participants were screened, and 10 participants were included. The rest of the screened participants did not match our inclusion criteria (n=7), could not commit to the study treatment protocol (n=2), or did not receive approval from their oncologist/physicians owing to their advanced condition (n=1). Of the 10 participants, seven successfully completed the study, two dropped out because of natural progression and exacerbation of their diseases, and one dropped out because of no improvement at the end of four treatments. Of the seven participants who completed the study, five were female and two were male. The median age was 66 years (range: 44–71 years). Four of the seven participants were diagnosed with breast cancer, and the other three were diagnosed with leukemia, non-Hodgkin’s lymphoma, and pancreatic cancer, respectively (Table 1).

**Table 1:** Demographic characteristics of the participants

**Participants characteristics**	**Participants (median age = 66 years)**	
	**n**	**%**
**Total**	**7**	**100**
**Gender**		
Female	5	71
Male	2	29
**Diagnosis**		
Leukemia	1	14
Non-Hodgkins lymphoma	1	14
Breast cancer	4	57
Pancreatic cancer	1	14

**Figure 1:**
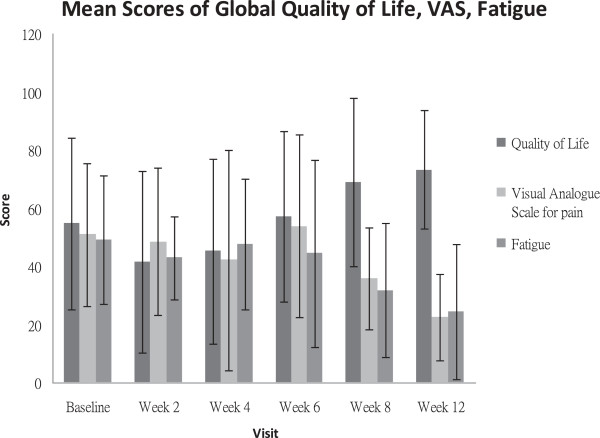
Mean scores of the general health status/QoL, VAS, and fatigue.

For the VAS, the percentage of improvement varied between 18% and 95% at the end of week 8 in five cases, whereas the pain worsened in two cases (Table 2). However, the mean raw score indicated an overall improvement in pain. The baseline mean raw score of 51±29.5 mm was reduced to 36±28.8 mm at the end of week 8 and to 23±20.3 mm at the end of week 12 (Table 3). For the EORTC QLQ, moderate or no improvement was noticed in the subscales of physical function, emotional function, and cognitive and social function. The percentage of improvement at the end of week 8 and the end of week 12 varied between 0% and 100% in individual cases. For the general health status/QoL, a subscale within the EORTC QLQ, the percentage of improvement varied between 20% and 100%. However, one participant reported decreases in QoL and general health status at the end of week 8 (−12%) and the end of week 12 (−25%) (Table 2). The mean raw score of general health and QoL improved with time. The baseline score of 55±22.0 increased to 66±22.9 at the end of treatment (week 8) and to 73±23.1 after follow-up (week 12), indicating an overall improvement in QoL (Table 3). Fatigue was the only symptom showing improvement, since the mean raw score of fatigue was reduced from 49.2±24.7 to 31.74±17.4 at the end of week 8 and to 24.44±14.8 at the end of week 12. Figure 1 illustrates the changes in the mean scores of the general health status/QoL, VAS, and fatigue with time.

**Table 2:** Percent improvements in the VAS, physical function, emotional function and QoL

**Participant**	**VAS**		**Physical function**		**Emotional function**		**General health (QoL)**	
	**End of week 8**	**End of week 12**	**End of week 8**	**End of week 12**	**End of week 8**	**End of week 12**	**End of week 12**	**End of week 12**
1	18	35	67	0	0	14	−13	−25
2	−36	NA	22	NA	0	NA	50	NA
3	−39	−21	8	15	12	38	20	20
4	33	NA	0	NA	100	NA	100	NA
5	78	78	22	44	−11	0	50	50
6	95	96	8	8	450	300	25	38
7	65	37	8	0	18	−45	25	−25

NOTE: Positive values for the VAS indicate improvement (decrease) of pain and negative values indicate worsening (increase) of pain. NA: Not available.

**Table 3:** Mean and SD scores of the VAS, fatigue, and general health status/QoL

	**Baseline Mean (SD)**	**Week 2 Mean (SD)**	**Week 4 Mean (SD)**	**Week 6 Mean (SD)**	**Week 8 Mean (SD)**	**Week 12 Mean (SD)**
**VAS**	51 (29.5)	49 (31.2)	42 (31.8)	54 (29.2)	36 (28.8)	23 (20.2)
**Fatigue**	49 (24.7)	42.3 (25.2)	48 (37.8)	44 (31.4)	32 (17.4)	24 (14.8)
**General QoL**	55 (22)	42 (14.43)	45 (22.4)	57 (32)	69 (22.9)	73 (23.1)

The results from the exploratory longitudinal regression analysis showed that there was a significant association (R^2^=83%, *P*=0.02) between the VAS pain score and the visit time, suggesting that the VAS score was significantly decreased (lower score means less pain) over time. For the QoL scales, the results were calculated for different subscales of the EORTC QLQ: physical function, emotional function, cognitive and social function, symptom scales, and global health status/QoL. No major change in the physical function, emotional function, and cognitive and social function scores was found, except in two patients. No major change in the symptom scales for any participants occurred, although slight negative changes in the symptom scales were noted in some participants. There was an overall positive change in all but one participant for the normal health status/QoL subscale by week 8. This positive change continued at the end of week 12 in all but one participant. Statistical analysis using the random mixed-effects model approach showed a significant association (R^2^=70%, *P*=0.02) between the general health status/QoL score and the visit time, suggesting that the general health status/QoL score was significantly increased with time. No significant side effects were reported by the participants throughout the study. However, one participant reported aggravation of pain, burning, and dizziness after the first treatment and during the second visit. These symptoms subsided on their own within 24 h and did not recur. No other side effects or adverse events were reported.

## Discussion

The results of this case series study indicate that the recruitment rate was acceptable (50%). Our best referral sources were cancer support groups and participants within our trial, who believed that they had benefits from participating in the study. Cancer support groups are often led by cancer survivors, and their meetings occur in a non-threatening atmosphere, function as a discussion forum for cancer patients and survivors, and provide much needed solace and guidance through the experiences of fellow members. Our recruitment was difficult because of our limited budget for advertisement. Furthermore, our recruitment team consisted of student workers, who were trained to screen and properly conduct the informed consent process to ensure trust between the investigating team and the participant. It remains unknown whether some participants did not sign up because of the lack of experience of the screening and recruiting personnel. Both financial and human resources are very crucial for successful recruitment.

Referral from oncologists was hindered by long distances between the patients’ homes and the study center. The oncologists were informed of the concurrent treatments of their patients. The cancer patients were reassured by the consent from their oncologists/physicians. All eligible patients with consent from their oncologists and physicians were willing to participate in our study. Thus, we plan to strengthen our communication with local oncologists to increase their recognition of our program and eventually aid the recruitment in future studies.

Positive changes with respect to both cancer pain and QoL were observed. A reduction in pain and an improvement in general health were noted at the end of week 8, which continued to improve (end of week 12) even after the treatment was discontinued.

Although these results are consistent with previous studies [30-32], no firm conclusions could be drawn from our findings because of the limitations inherent in a case series design including a small sample size and lack of a control group. Furthermore, we were unable to obtain complete follow-up data of all participants and interpret the data toward any specific type of cancer pain or QoL issues. It is also recommended to set a minimum score for the QoL scale as an eligibility criterion, to avoid a ceiling effect when assessing differences in QoL measures.

## Conclusion

In this pilot study, the results of the EORTC QLQ-C3 and VAS showed that acupuncture might be beneficial for reducing pain and improving QoL in cancer patients.

## Abbreviations

CAM: Complementary and alternative medicine; EORTC QLQ: European Organization for Research and Treatment of Cancer Quality of Life Questionnaire; VAS: Visual analog scale; QoL: Quality of life.

## Competing interests

The authors declare that there is no conflict of interest with any financial organization regarding material discussed in this manuscript.

## Authors’ contributions

SPV, JL, EH, CKH, and CH designed the study and wrote the manuscript. SPV, JL, EH, and CH performed the treatments. SPV, YH, and JM analyzed the data. All authors read and approved the final manuscript.
